# A novel inversion in the chloroplast genome of marama (*Tylosema esculentum*)

**DOI:** 10.1093/jxb/erw500

**Published:** 2017-01-31

**Authors:** Yunsoo Kim, Christopher Cullis

**Affiliations:** 1 Department of Biology, Case Western Reserve University, Cleveland, OH 44106, USA

**Keywords:** Basal legume, chloroplast genome sequence, intraspecific variation, marama, unique inversion

## Abstract

*Tylosema esculentum* (marama bean) is being developed as a possible crop for resource-poor farmers in arid regions of Southern Africa. As part of the molecular characterization of this species, the chloroplast genome has been assembled from next-generation sequencing using both Illumina and Pac-Bio data. The genome is of typical organization with a large single-copy region and a small single-copy region separated by a pair of inverted repeats and covers 161537 bp. It contains a unique inversion not present in any other legumes, even in the closest relatives for which the complete chloroplast genome is available, and two complete copies of the *ycf1* gene. These data extend the range of variability of legume chloroplast genomes. The sequencing of multiple individuals has identified two different chloroplast genomes which were geographically separated. The current sampling is limited so that the extent of the intraspecific variation is still to be determined, leaving open the question of legume chloroplast genomes adapted to particular arid environments.

## Highlight

The chloroplast genome of a basal legume has been assembled from next-generation sequence information. This genome, which has a unique inversion, an additional copy of the *ycf1* gene, and sequence variation among individuals, has been characterized.

## Introduction

The marama bean [*Tylosema esculentum* (Burchell) Schreiber] is a wild tuber-producing and non-nodulating basal legume that grows in arid regions of Southern Africa and has been identified as an orphan crop (National Research Council, 1996). The species is being investigated as a possible crop with the potential to contribute to the food security of resource-poor farmers (Chimwamurombe, 2008; Nepolo *et al.*, 2009).

Marama is one of the basal legumes in the *Caesalpinioideae*, in the tribe *Cercideae*, which is subdivided into two subtribes, the *Cercidinae* and *Bauhiniinae. Tylosema* was originally classified within in the genus *Bauhinia* (Hao *et al.*, 2003) but then was reclassified as a separate clade within the *Bauhiniinae* (Wunderlin, 2010). The relationships between the species have been subject to both palynological and molecular analyses, the latter especially with the use of chloroplast markers such as the *matK* gene (Hao *et al.*, 2003; Banks *et al.*, 2013).

The characterization of both the genetic and phenotypic variability is an important component of the domestication process. Earlier studies on marama using various DNA marker systems including rDNA (Nepolo *et al.*, 2010) and simple sequence repeats/microsatellites (SSRs) (Takundwa *et al.*, 2010) have demonstrated that the marama bean has a high intrapopulation diversity and a low interpopulation diversity. In addition, the chloroplast *mat*K gene was used to construct a phylogenetic tree including marama and shown to be useful for determining levels of genetic variation (Takundwa *et al.*, 2012).

The organization of the plastome is highly conserved in most flowering plants (Jansen and Ruhlman, 2012), with the structure usually having a large and a small single-copy region separated by a pair of inverted repeats. The chloroplast genomes in the legumes are very varied. Two of the major variants are the loss of one copy of the inverted repeat and a 50 kb inversion (Doyle *et al.*, 1996). In addition, lupin has a 36 kb inversion within this 50 kb inversion (Martin *et al.*, 2014). This considerable atypical plastome variation makes them an emerging model system to investigate aspects of plastome evolution (Dugas *et al.*, 2015). Although there are >60 complete chloroplast genomes reported from the legumes, most are from the Papilionoideae. The few from other clades includes that for *Acacia ligulata* in the *Mimosoideae* (Williams *et al.*, 2015) and one from the *Caesalpinioideae* (for *Cercis canadensis*), so this study adds knowledge on the variation of the chloroplast genome for the basal legumes.

High-throughput next-generation sequencing has been applied to marama with data from both the short read Illumina platform as well as the long read PacBio platform. These data have been used to assemble the complete marama chloroplast genome. The overall organization is as expected, with large and small single copy regions separated by a pair of inverted repeats. As expected from the taxonomic position of this species as a basal legume, the large (50 kb) inversion is absent from the marama chloroplast genome as it is from the most closely related clades, namely the Caesalpinioid crown clade, ADA clade, Swartzoid clade, and the Cladrastis clade (Martin *et al.*, 2014). However, a major structural difference, a unique inversion of 7500 bp which is absent from all the other legume chloroplasts sequenced to date, has been identified.

In this study, the complete chloroplast genome of marama has initially been assembled and characterized for a single individual. Following the assembly of the first chloroplast genome, next-generation sequencing data from eight other individuals were aligned to this chloroplast assembly. These data have identified a second form of the chloroplast genome. All of these eight individuals, from a collection at the University of Pretoria farm, had identical chloroplast genomes that differed from the first assembly in 94 single nucleotide polymorphisms (SNPs) and 77 indels, and is consistent with different chloroplast genomes being distributed geographically.

## Materials and methods

### Plant material

Nine different individuals were sampled. Six were growing long term at the University of Pretoria Farm. Three seeds, two collected from different plants at the UP farm, and one from Namibia were germinated in sand and grown in a greenhouse. DNA from all the samples was extracted from young leaves.

### DNA extraction and estimation

Young leaves from the growing tip of each of the nine plants were collected and total DNA extracted using the Qiagen Plant miniprep kit. The leaves were ground in a mortar with acid-washed sand to ensure cell breakage. All other steps were as per the manufacturer’s protocol. The DNA yield and quality was determined both by using a NanoDrop spectrophotometer and by electrophoresing 5 μl of the extracted DNA on a 1% agarose Tris/borate/EDTA gel at 105 V for 1 h, using ethidium bromide to visualize the DNA.

### High-throughput sequencing

The nine DNA samples (total leaf DNA containing nuclear, chloroplast, and mitochondrial DNAs) were sent to the Génome Québec Innovation Centre for sequencing. All the steps for the next-generation sequencing were performed at the Innovation Center. The DNA from all nine samples was fragmented to an average size of 308 bp, barcoded, and loaded on a single lane of an Illumina HiSeq 2000 PE100. The total number of bases for each of the samples varied from 3.4 Gb to 4.3 Gb. The portion of the reads that aligned to the chloroplast genome in these data sets varied between 5.8% and 15.3%, representing a coverage of between 1700× and 4500× of the chloroplast genomes (Supplementary Data S1). One sample (the one from Namibia) was also sequenced in five PacBio SMRT cells, giving a total of 1.78 Gb of sequence. Of this sequence, 1.66 Gb was in reads >3 kb (Supplementary Data S1). The proportion of reads that align to chloroplast sequences in this data set (4.8%) was similar to that for the Illumina sequencing data.

### Chloroplast genome assembly

Using the i-Plant Discovery Environment (now named Cyverse), the paired end reads were aligned to 29 chloroplast genomes (given in Supplementary Table S1 at *JXB* online) using bowtie2 (Langdon, 2015). The mapped reads were outputted to fastq files, which were interlaced and then concatenated. Duplicates were removed using prinseq-lite.

The mapped reads were assembled *de novo* using ABySS (http://www.bcgsc.ca/platform/bioinfo/software/abyss). The ABySS assembly gave contigs up to 73 kb in length, which were used for elongating and scaffolding. DBG2OLC (Ye *et al*., 2014) was used for elongating contigs using the Illumina contigs as anchors to build overlap graphs using PacBio reads. The results still did not result in a complete contiguous chloroplast assembly. Therefore, the PacBio reads were converted into a Blast database in Genious version 9 and used to identify reads that crossed the remaining gaps in the assembly. The consensus sequences for each region were extracted and used to generate a version of the complete assembled chloroplast. All the Illumina reads from the same sample used for the PacBio sequence were aligned to the new assembly and the assembled sequence corrected for any ambiguities identified. The procedure was reiterated until there were no ambiguities in the alignment of the Illumina reads. This was considered the final sequence of the chloroplast of this individual marama plant. The junctions of the inverted repeat regions were mapped using the PacBio reads across the putative junctions, with the exact position of the junction being readily apparent by a break in the sequence between the two ends of the inverted repeat.

The other eight Illumina genomic read data sets (all from the University of Pretoria plants) were then aligned to this chloroplast assembly (Supplementary data S1). All of these eight samples were identical to each other, but different from that for the Namibian sample. A second chloroplast molecule, typical of the South African samples, was also constructed. The genome from the initial assembly has been submitted to GenBank.

The PCRs were performed to verify the inversion. Pairs of primers were designed, using Primer 3, that would amplify across the two ends of the inversion if linear. The primers were: primer 1 Forward, TACCTAACATATTTTTTTTA; primer 1Reverse, AATCGCCTTT CCTATTCTT; product size: 1101 bp; primer 2 Forward, CATCGGTCCACACAGTTGTC; primer 2Reverse, CCCTGTA GGAATCGGATGAA; product size: 1162 bp.

The amplifications were performed in 20 μl using Promega GoGreenTaq polymerase. The denaturation was performed at 94 °C for 2 min followed by 30 cycles of 94 °C for 20 s, 55 °C for 30 s, and 72 °C for 75 s, and a final 72 °C for 5 min. The products were separated on a 1.5% agarose gel in Tris–Borate–EDTA at 100 V.

### Chloroplast sequence analysis

Both assembled chloroplast molecules were annotated using CPGAVAS (http://www.herbalgenomics.org/0506/cpgavas/analyzer/home) and DOGMA (http://dogma.ccbb.utexas.edu/cgi-bin/new_user.cgi). Both programs gave essentially the same set of genes and positions. Both of the chloroplast molecules were compared with various other published chloroplast genome sequences using Blastn, as well as being compared with each other.

### Phylogenetic analysis

The phylogenetic tree was determined using a set of 29 protein-coding genes that are present in all the families (*psb*, *matK*, *atpA*, *atpF*, *atpF*, *rpoB*, *psbD*, *psbC*, *psaB*, *psaA*, *ndhK*, *psbG*, *ndhC*, *atpE*, *atpB*, *rbcL*, *accD*, *cemA*, *petA*, *rpl20*, *psbB*, *petB*, *rpoA*, *ndhH*, *ndhA*, *ndhI*, *ndhG*, *ndhE*, and *ndhD*) from seven legumes, and *Arabidopsis thaliana* as the outgroup. The concatenated protein set was aligned in KAlign (www.ebi.ac.uk/Tools/msa/kalign) and the alignment subsequently analyzed using MrBayes (Huelsenbeck and Ronquist, 2001; Ronquist and Huelsenbeck, 2003) in the Genious platform.

## Results

### Organization, gene content, and characteristics of the *T. esculentum* chloroplast

**Fig. 1.**
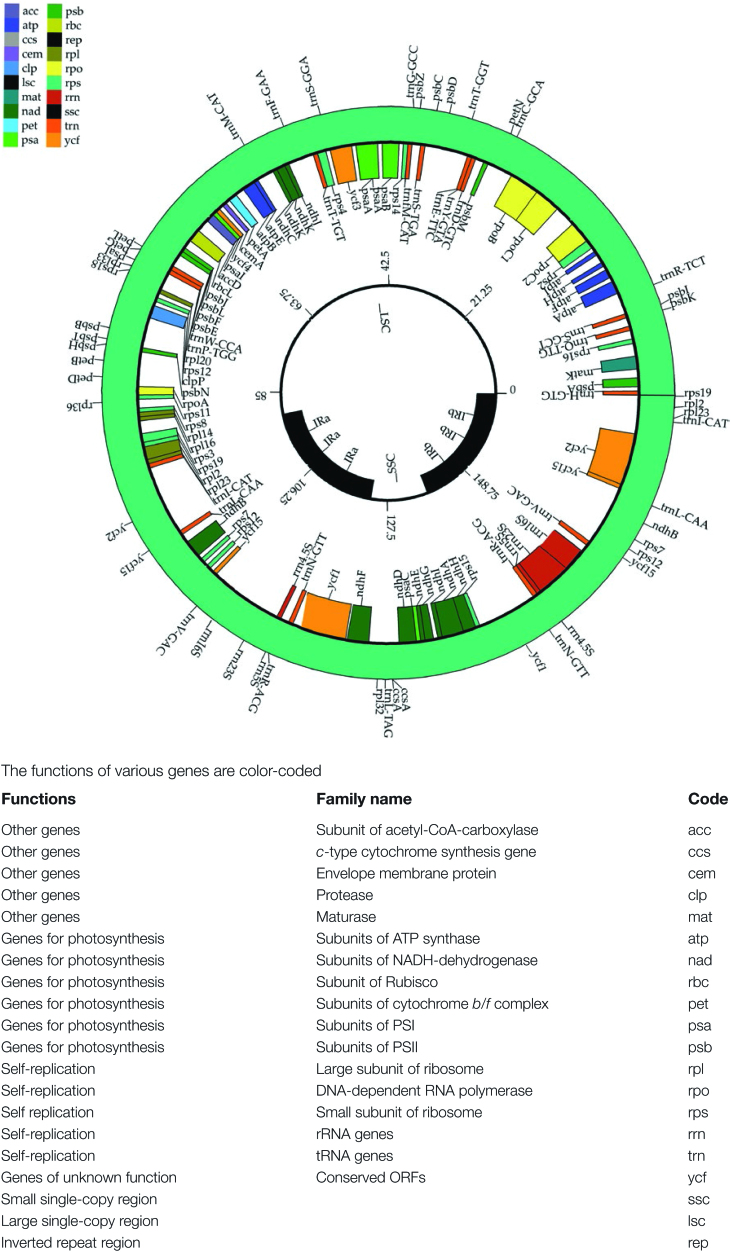
Circular gene map of the *Tylosema esculentum* (Genistoid; Fabaceae) plastid genome. Genes are represented with boxes inside and outside the first circle to indicate a clockwise or counterclockwise transcription direction, respectively. Genes belonging to different functional groups are color coded. The locations of the different main plastomic regions (inverted repeats, large single copy, and small single copy) are indicated in the inner circle. The molecule was drawn through the analysis site http://www.herbalgenomics.org/0506/cpgavas/analyzer/home.

The complete chloroplast genome was determined for nine different individuals. The first compilation was from an individual collected in Namibia and was used as the standard sequence since DNA sequence data from both Illumina and PacBio sequencing were available. The initial build resulted in a circular chloroplast genome of 161537 bp, that is organized in the expected form of a pair of inverted repeats separated by a large single-copy region and a small single-copy region (Fig. 1). This sequence and its annotation have been deposited in GenBank (accession no. KX792933). The circularity of the chloroplast molecule was confirmed using a synthetic genome crossing the end of the inverted repeat into the large single-copy region and aligning this to both the Illumina reads and the PacBio reads. In both cases, the number of reads across the junction was the same as the number for either end independently, confirming the circularity. It was also independently confirmed by PCR amplification using a pair of primers across the junction, and the correct sized fragment was amplified.

### Physical features of the *T. esculentum* chloroplast genome

The complete *T. esculentum* chloroplast genome was shown to be a typical circular molecule which encodes 161537 nucleotides, comprised of the expected four regions, the large single-copy (LSC), the small single-copy (SSC), and the two inverted repeat regions (IRa and IRb) (Fig. 1). These four regions of the circular chloroplast genome occupy 86112 bp for LSC, 13630 bp for SSC, and 61806 bp (30903 bp each) for the set of inverted repeat regions (Table 1). The chloroplast genome is larger than that of *Acacia*, 158724 bp (Williams *et al.*, 2015), and *Cercis*, 158995 bp (https://www.ncbi.nlm.nih.gov/nuccore/KF856619.1), but contains a larger inverted repeat region and a shorter SSC region than either of these two species. The chloroplast genome sizes in legumes range from 175489 nucleotides for *Inga leiocalycina* to 120289 nucleotides for *Lathyrus odoratus* cultivar Cupani. All the smaller legume chloroplast genomes have lost one copy of the inverted repeat region.

**Table 1.** Summary of chloroplast genome characteristics of marama

Total size (bp)	161537
LSC size in bp	86113
SSC size in bp	13630
IR length in bp	30897
Size of coding regions in bp	101241
Size of protein-coding regions in bp	80218
Size of rRNA in bp	10282
Size in bp of tRNA	10741
Size in bp of intergenic regions	60296
No. of different genes	125
No. of different protein-coding genes	79
No. of different tRNA genes	30
No. of different rRNA genes	4
No. of different genes duplicated by IR	17
No. of different genes with introns	22
Overall % GC content	36.13%
% GC content in protein-coding regions	37.5%
% GC content in IGSs	31.16%
% GC content in rRNA	54.6%
% GC content in tRNA	43.4%

The chloroplast of marama encodes 79 unique protein-coding genes, of which eight are duplicated in the inverted repeat region, 30 unique tRNA genes, of which seven are duplicated in the IR region, and four unique rRNA genes. The categories of genes identified and their functions are given in Table 2. Eleven-protein coding genes that contained introns are listed in Table 3. None of these genes appear to be pseudogenes.

**Table 2.** Coding regions of the marama chloroplast

**Family name**	**List of genes**
rRNAs	16S (*rrn16*) (×2), 23S (*rrn23*) (×2) 4.5S (*rrn4.5*) (×2), 5S (*rrn5*) (×2)
tRNAs	tRNA-His(GTG), tRNA-Lys(TTT), tRNA-Gln(TTG), tRNA-Ser(GCT), tRNA-Thr(CGT), tRNA-Arg(TCT), tRNA-Cys(GCA), tRNA-Asp(GCT), tRNA- Tyr(GTA), tRNA-Glu(TTC), tRNA-Thr(GGT), tRNA-Ser(TGA), tRNA-Gly(GCC), tRNA-Met(CAT), tRNA-Ser(GGA), tRNA-Thr(TGT), tRNA-Leu(TAA), tRNA-Phe(GAA), tRNA-Ile(AAT), tRNA-Met(CAT), tRNA-Trp(CCA), tRNA-Pro(TGG), tRNA-Met(CAT) (×2), tRNA-Leu(CAA) (×2), tRNA-Val(GAC) (×2), tRNA-Glu(TTC) (×2), tRNA-Ala(TGC) (×2), tRNA-Arg(ACG) (×2), tRNA-Asn(GTT) (×2), tRNA-Leu(TAG)
Small subunit of ribosome	*rps2*, *rps3*, *rps4*, *rps7* (×2), *rps8*, *rps11*, *rps12* (×2, part), *rps14*, *rps15*, *rps16*, *rps18*, *rps19*
Large subunit of ribosome	*rpl2* (×2), *rpl14*, *rpl16*, *rpl20*, *rpl23* (×2), *rpl32*, *rpl33*, *rpl36*
RNA polymerase	*rpoA*, *rpoB*, *rpoC1*, *rpoC2*
NADH-dehydrogenase	*ndhA*, *ndhB* (×2), *ndhC*, *ndhD*, *ndhE*, *ndhF*, *ndhG*, *ndhH*, *ndhI*, *ndhJ*, *ndhK*
PSI	*psaA*, *psaB*, *psaC*, *psaI*, *psaJ*, *ycf3* (×2)
PSII	*lhbA*, *psbA*, *psbC*, *psbD*, *psbE*, *psbF*, *psbH*, *psbI*, *psbJ*, *psbK*, *psbL*, *psbM*, *psbN*, *psi-psbT*, *psbT*
Cytochrome *b*/*f*	*petA*, *petB*, *petD*, *petG*, *petL*, *petN*
ATP synthase	*atpA*, *atpB*, *atpE*, *atpF*, *atpH*, *atpI*
Rubisco	*rbcL*
Subunit of acetyl-CoA-carboxylase	*accD*
Others	*matK*, *clpP*, *cemA*, *ccsA*
Unknown function ORFs	*ycf1* (×2), *ycf2* (×2), *ycf4*, *ycf15*, *ycf68*

**Table 3.** The lengths of introns and exons for the splitting genes

**Gene**	**Strand**	**Start**	**End**	**Exon I**	**Intron I**	**Exon II**	**Intron II**	**Exon III**
*atpF*	–	12120	13437	148	709	461		
*rpoC1*	–	21208	24065	437	800	1621		
*psaA*	–	41327	43468	1788	30	324		
*ycf3*	–	44330	46381	127	716	230	829	150
*accD*	–	60367	61305	238	120	581		
*clpP*	–	71533	72885	299	837	217		
*rpl2*	–	86463	87955	394	632	467		
*ndhB*	–	96955	99163	870	586	753		
*ndhA*	–	126748	129120	552	1263	558		
*ndhB*	+	148488	150699	867	589	756		
*rpl2*	+	159696	161191	391	635	470		

Consistent with the previous observations in the *Leguminosae*, the *rpl22* gene is absent from the *T. esculentum* plastid genome following an ancient transfer to the nuclear genome (Gantt *et al.*, 1991). In those legume species that have retained their inverted repeat, the inverted repeat runs ~450 bp into the *ycf1* gene (Williams *et al.* 2015). This is true for species closely related to marama including *Acacia ligulata* (437 bp), *Millettia pinnata* (446 bp) (Williams *et al.*, 2015), and *Cercis canadensis* (488 bp) (GenBank accession no. KF856619.1) genomes. However, the expanded inverted repeat in marama has resulted in the inclusion of the complete *ycf1* gene, which now is present intact in two copies.

### Unique inversion

**Fig. 2.**
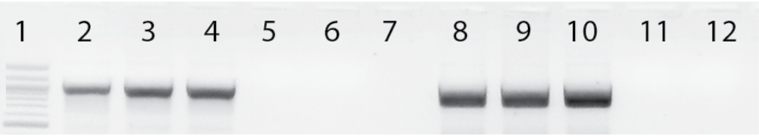
Amplification across the two ends of the inversion confirming the arrangement of the sequences as assembled. DNAs from three different marama plants and from lupin as a control were used. Lane 1, New England Biolabs 100 bp ladder (bands visible at 1517, 1200, 1000, 900, 800, 700, 600, and 500 bp); lane 2, marama DNA; lane 3, marama DNA; lane 4, marama DNA; lane 5, lupin DNA lane 6, water; lane 7, blank; lane 8, marama DNA; lane 9, marama DNA; lane 10 marama DNA; lane 11, lupin DNA; lane 12, water. The products in lanes 2–6 were amplified with primers 1Forward and 1Reverse, while those in lanes 8–12 were amplified with primers 2Forward and 2Reverse.

**Fig. 3.**
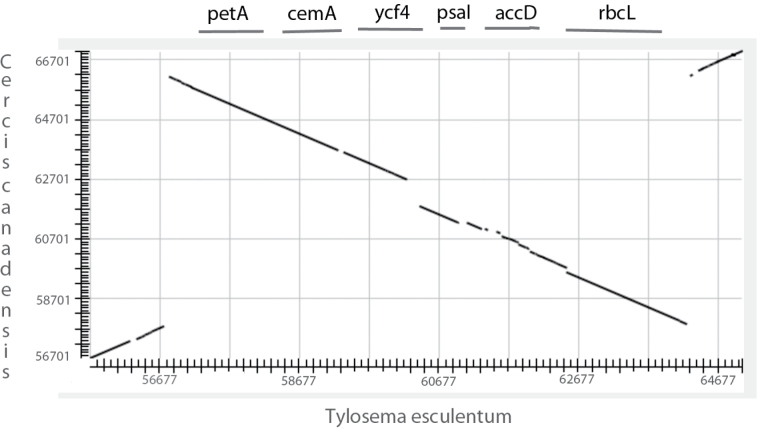
Alignment of the sequences from marama and *Cercis canadensis* chloroplast genomes covering the inversion region. The respective regions of the chloroplasts were aligned using Blastn (megablast) for high similarity. Note, not only the inversion but the differences in sequences across this region. The organization of the protein-coding genes is shown. The axes are labeled with the nucleotide positions in the chloroplast molecules, for marama the new genome and for *Cercis* the genome available at NCBI (KF856619.1).

The marama chloroplast genome appears to have a unique inversion, included in the LSC region, among the legumes. The inverted region is 7479 bp and includes the six genes *rbcL*, *accD*, *psaI*, *ycf4*, *cemA*, and *petA*. Five of the six genes associated with the inversion (all except *rbcL*) starting with *accD* are adjacent to the end of the 50 kb inversion in lupin. A major variation in the marama chloroplast genome with respect to other legumes is the altered location of the *rbcL* gene. The presence of the inversion has been confirmed using PCR with two sets of primers designed to amplify across the junctions of the inversion in marama (Fig. 2). The inversion region between the chloroplast genomes of marama and its closest relative among the species with a complete chloroplast genome sequence, *C. canadensis*, is shown in Fig. 3. When compared with all the other legume chloroplast genomes, this specific inversion is unique to marama. It also does not appear to be present in any of the other comparisons with non-legume chloroplasts that have been performed. An 11 kb fragment spanning the inversion was aligned to the complete chloroplast genomes of 11 non-legume dicotyledon species (*Artemisia frigida*, *Brassica juncea*, *Camellia pubicosta*, *Cucumis sativus*, *Gossypium bickii*, *Macadamia integrifolia*, *Mesembryanthemum crystallinum*, *Nicotiana tabacum*, *Populus euphratica*, *Prunus persica*, and *Solanum tuberosum*) and one monocot species (*Hordeum vulgare*).

The break point for the 50 kb inversion clade in the legumes is between the *accD* gene and the *rbcL* gene (Martin *et al.*, 2014). This break point results in the *accD* gene not being moved, while the inversion in marama includes the *accD* gene in the inverted segment. Therefore, this region of the chloroplast genome in legumes is involved in rearrangements more frequently than in other plants, where it appears to be collinear in the other dicotyledon species and the single monocotyledon species. No obvious structural elements were detected at the junctions of the inversion such as direct or inverted repeats across regions where the ends of the inversion should reside. The break points do not disrupt any genes.

### Intraspecific variation in chloroplast sequence

The chloroplast sequence diversity in the legumes has mainly been concentrated on the interspecific differences, with few data on intraspecific variation. The data reported here identify two distinct chloroplast genomes in *T. esculentum*. These two forms have >200 differences between them. Most of these differences are SNPs although there are also some indels. The positions of the 94 SNPs (three of which occur within the inverted repeats) are given in Supplementary Table S2. The data only represent a small sampling of the geographical diversity of marama. The origin between the plants growing at the University of Pretoria farm is obscure and they may be related although they are phenotypically diverse. Additional sampling is currently underway, across many regions of the range, to determine the extent and distribution of the intraspecific chloroplast variation.

The SNP variants do not affect the coding capacity of the marama chloroplast genome. Seventy of the 94 SNPs are in non-coding regions. The effects of the remaining 24 SNPs in coding regions were tested by translating the two alternative sequences and comparing the two resulting amino acid sequences using blast. In every case, the two alternatives resulted in the same protein sequence and therefore the polymorphisms do not affect the protein sequences.

Intraspecific variation in chloroplast genomes has been described in legumes, initially through changes in restriction enzyme recognition sites (Lavin *et al.*, 1991) and more recently through whole-genome sequencing (Lei *et al.*, 2016). The whole-genome sequence analysis for *Astragalus membranaceus* mainly identified insertion/deletions in five hypervariable regions. In rice Wei Tong *et al.* (2016), have identified 3677 variations across 383 rice accessions from diverse origins. However, the level of variation between any two varieties is not explicitly identified so cannot be compared with the level of variation identified in marama here.

### Taxonomic relationships based on complete chloroplast genome sequences

**Fig. 4.**
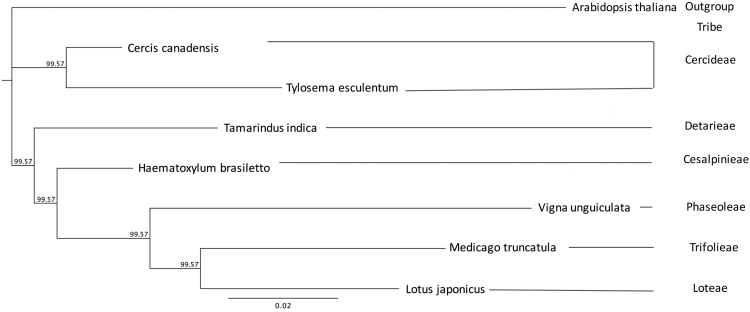
Phylogenetic relationships. Twenty-nine proteins (psb, matK, atpA, atpF, atpF, rpoB, psbD, psbC, psaB, psaA, ndhK, psbG, ndhC, atpE, atpB, rbcL, accD, cemA, petA, rpl20, psbB, petB, rpoA, ndhH, ndhA, ndhI, ndhG, ndhE, and ndhD) were identified from the available chloroplast genomes of *Arabidopsis thaliana* (AP000423.1), *Cercis canadensis* (KF856619.1), *Haematoxylum* brasiletto (NC_026679), *Lotus japonicus* (NC_002694.1), *Medicago truncatula* (NC_003119.6), *Tamarindus indica* (NC_026685), *Tylosema esculentum* (KX792933), and *Vigna unguiculata* (NC_018051.1) in NCBI and concatenated. The protein sets were aligned in KAlign and then their relationship tree determined by MrBayes.

The taxonomic relationships within the legumes are well established, especially for those species of agronomic importance, using chloroplast genomes, or subsets of common genes, as the basis for comparison (Martin *et al.*, 2014; Lei *et al.*, 2016). Here, the relationship between marama and other chloroplast genomes has been determined using both the complete chloroplast genomes and a subset of the protein-coding regions. The phylogenetic tree shown in Fig. 4 was determined using a set of 29 protein-coding genes (*psb*, *matK*, *atpA*, *atpF*, *atpF*, *rpoB*, *psbD*, *psbC*, *psaB*, *psaA*, *ndhK*, *psbG*, *ndhC*, *atpE*, *atpB*, *rbcL*, *accD*, *cemA*, *petA*, *rpl20*, *psbB*, *petB*, *rpoA*, *ndhH*, *ndhA*, *ndhI*, *ndhG*, *ndhE*, and *ndhD*) that are present in all the families. The concatenated protein set was aligned in KAlign (www.ebi.ac.uk/Tools/msa/kalign) and the alignment subsequently analyzed using MrBayes (Huelsenbeck and Ronquist, 2001; Ronquist and Huelsenbeck, 2003) in the Genious platform. As expected, *C. canadensis* was the most closely related species to *T. esculentum*, with the other species following the previously determined pattern of relationships (Martin *et al.*, 2014) in this analysis.

## Discussion

The assembly of the chloroplast genome from *T. esculentum* has highlighted differences in the organization of a member of the basal legumes. The major differences that have been identified include the presence of a specific inversion that has not been found in any of the legume chloroplasts sequenced to date, an expansion of the inverted repeat to include a complete copy of the *ycf1* gene, and the identification of at least two different chloroplast genomes. It would be interesting to characterize this region in Bauhinia species to determine if the inversion is specific to the *Tylosema* or is present in all the members of this clade.

Since the emergence of the Fabaceae, there has been loss of five different chloroplastic genes: *accD*, *psaI*, *rpl23*, *rps16*, and *ycf4* (Jansen *et al.*, 2007; Magee *et al.*, 2010). In *Tylosema*, all of these genes are present. As would be expected, all of these genes are also present in the *C. canadensis* chloroplast genome, which is the most closely related species with a complete chloroplast genome available.

The intraspecific variation in the marama chloroplast genome is being investigated through whole-genome sequencing of individuals from various regions of Namibia to determine whether there are more examples of intraspecific variation and whether any such variants are geographically isolated. This next set of data will illuminate the extent of the intraspecific variation. Since the geographical origin of the samples being sequenced is known, the new data should also identify any gene flow and distribution of seeds across the region. The presence of specific chloroplast genomes in particular regions would suggest that the seeds are not widely distributed, while a mixture of genomes across all regions would indicate a wide distribution of the seeds and/or pollen.

## Supplementary Material

Supplementary Data
