# Supplementary material for: A novel inversion in the chloroplast genome of marama (Tylosema esculentum)
Source: J Exp Bot. 2017 Jan 31;68(8):2065–72. doi: 10.1093/jxb/erw500 (PMC5429017; doi:10.1093/jxb/erw500)
Supplement: Supplementary Data [file erw500_Supplementary_Data.zip › supplementary_data_S1_tables_S1_S2.pdf]

A novel inversion in the chloroplast genome of marama (*Tylosema esculentum*)

Yunsoo Kim and Christopher Cullis

Supplementary data.

The alignment statistics from Bowtie2 for the Illumina reads against the final assembled chloroplast genome.

```
179470509 reads (100.00%) were paired; of these:
  168506805 (93.89%) aligned concordantly 0 times
  5842266 (3.26%) aligned concordantly exactly 1 time
  5121438 (2.85%) aligned concordantly >1 times
  ----
  168506805 pairs aligned concordantly 0 times; of these:
    99221 (0.06%) aligned discordantly 1 time
  ----
  168407584 pairs aligned 0 times concordantly or discordantly; of these:
    336815168 mates make up the pairs; of these:
      336495371 (99.91%) aligned 0 times
      103582 (0.03%) aligned exactly 1 time
      216215 (0.06%) aligned >1 times

6.25% overall alignment rate
```

Chloroplast reads from the PacBio sequence dataset:

The proportion of reads that align to chloroplast sequences in this data set is 4.8%.

A novel inversion in the chloroplast genome of marama (*Tylosema esculentum*)

Yunsoo Kim and Christopher Cullis

Supplementary Table 1. Chloroplast genomes, and their accession numbers, used for extracting reads from marama next gen sequencing.

Species	Accession number
Acacia ligulata	LN555649.1
Arabidopsis thaliana	NC_000932.1
Cicer arietinum	NC_011163.1
Corynocarpus laevigata	NC_014807.1
Eucalyptus grandis	NC_014570.1
Glycine max	NC_007942.1
Glycyrrhiza glabra	NC_024038.1
Gossypium hirsutum	NC_007944.1
Haematoxylum brasiletto	KJ468097.1
Helianthus annuus	DQ383815.1
Humulus lupulus	NC_028032.1
Lathyrus sativus	NC_014063.1
Libidibia coriaria	KJ468095.1
Liquidambar formosana	NC_023092.1
Lotus japonicus	NC_002694.1
Lupinus luteus	KC695666.1
Medicago truncatula	NC_003119.6
Millettia pinnata	NC_016708.2
Panax notoginseng	NC_026447.1
Pentactina rupicola	NC_016921.1
Phaseolus vulgaris	NC_009259.1
Pisum sativum	NC_014057.1
Populus trichocarpa	NC_009143.1
Prosopis glandulosa	KJ468101.1
Prunus persica	NC_014697.1
Trifolium subterraneum	NC_011828.1
Vigna radiata chloroplast	NC_013843.1
Vitis vinifera	NC_007957.1
Zea mays chloroplast	NC_001666.2

A novel inversion in the chloroplast genome of marama (*Tylosema esculentum*)

Yunsoo Kim and Christopher Cullis

Supplementary table 2. The positions of the identified SNPs within the chloroplast genome.

Genome Position	Reference Allele	Alternate Allele	Presence in gene
1	T	C	n
3	C	T	n
4	T	A	n
2237	A	G	n
3172	A	G	MatK
3696	G	T	n
3817	T	A	n
4694	C	A	n
5052	G	T	n
6295	G	T	n
6435	C	A	n
6749	C	A	n
7025	G	T	n
7969	C	T	n
10430	T	A	n
10431	A	C	n
11660	G	A	in atpA
15831	A	G	n
17119	T	C	rpoC2
17499	T	C	rpoC2
20769	T	G	rpoC2
23154	A	T	n
24858	G	A	rpoB
26559	T	C	rpoB
27162	A	C	rpoB
27371	A	G	n
28116	C	A	n
28666	T	C	n
28779	G	T	n
36942	A	G	n

37257	A	G	n
38975	C	T	n
41243	A	G	psaB
42587	C	T	psaA
47947	T	A	n
47975	A	T	n
48447	T	G	n
48631	T	G	n
49457	G	A	n
49808	T	C	n
52550	T	C	n
53758	C	A	n
55953	T	C	atpB
59237	C	A	n
59911	A	C	n
60379	C	T	accD
61858	A	C	n
61866	G	A	n
62209	C	T	n
62213	C	T	n
68613	A	C	n
68683	C	T	n
68988	A	C	n
72090	T	C	n
73652	A	G	n
77089	G	T	n
80191	A	C	n
80845	A	G	rpoA
82340	C	T	n
84542	C	T	n
84737	G	T	n
84997	T	A	n
85196	T	G	rps3
85632	C	T	rps3
110050	T	A	n
110051	A	T	n
110052	T	A	n
117730	C	A	ndhF
117839	A	G	ndhF
119473	G	T	n

119479	A	C	n
119732	C	A	n
119964	G	A	n
120929	A	T	n
121033	A	G	n
121127	G	A	n
122110	C	A	n
122836	A	C	ndhD
123509	T	G	ndhD
123734	A	T	ndhD
125353	C	T	ndhG
125926	G	A	n
126055	A	T	n
126149	G	T	n
126236	A	G	ndhI
126967	T	C	ndhA
127576	T	G	n
128069	A	C	n
128495	T	C	n
129615	C	T	n
129834	A	T	ndhH
137599	A	T	n
137600	T	A	n
137601	A	T	n
